# Cross-border comparison of antimicrobial resistance (AMR) and AMR prevention measures: the healthcare workers’ perspective

**DOI:** 10.1186/s13756-019-0577-4

**Published:** 2019-07-22

**Authors:** J. Keizer, L. M. A. Braakman-Jansen, S. Kampmeier, R. Köck, N. Al Naiemi, R. Te Riet-Warning, N. Beerlage-De Jong, K. Becker, J. E. W. C. Van Gemert-Pijnen

**Affiliations:** 1 Department of Psychology, Health and Technology, Centre for eHealth and Wellbeing Research, University of Twente, P.O. Box 217, 7500AE Enschede, The Netherlands; 2 Institute of Hygiene, University Hospital Münster, Münster, Germany; 3 Institute of Medical Microbiology, University Hospital Münster, Münster, Germany; 4 Institute of Hospital Hygiene Oldenburg, Oldenburg, Germany; 5 Department of Infection Prevention, Hospital Group Twente, Almelo/Hengelo, Netherlands; 6 LabMicTA, Hengelo, Netherlands

**Keywords:** Antimicrobial resistance (AMR), Healthcare worker, Infection control, Cross-border, Prevention, Multidrug-resistant microorganisms, Euroregion, Germany, Netherlands

## Abstract

**Background:**

Cross-border healthcare may promote the spread of multidrug-resistant microorganisms (MDRO) and is challenging due to heterogeneous antimicrobial resistance (AMR) prevention measures (APM). The aim of this article is to compare healthcare workers (HCW) from Germany (DE) and The Netherlands (NL) on how they perceive and experience AMR and APM, which is important for safe patient exchange and effective cross-border APM cooperation.

**Methods:**

A survey was conducted amongst HCW (*n* = 574) in hospitals in DE (*n* = 305) and NL (*n* = 269), using an online self-administered survey between June 2017 and July 2018. Mann-Whitney U tests were used to analyse differences between answers of German and Dutch physicians (*n* = 177) and German and Dutch nurses (*n* = 397) on 5-point Likert Items and Scales.

**Results:**

Similarities between DE and NL were a high awareness about the AMR problem and the perception that the possibility to cope with AMR is limited (30% respondents perceive their contribution to limit AMR as insufficient). Especially Dutch nurses scored significantly lower than German nurses on their contribution to limit AMR (means 2.6 vs. 3.1, *p* ≤ 0.001). German HCW were more optimistic about their potential role in coping with AMR (p ≤ 0.001), and scored higher on feeling sufficiently equipped to perform APM (*p* ≤ 0.003), although the mean scores did not differ much between German and Dutch respondents.

**Conclusions:**

Although both German and Dutch HCW are aware of the AMR problem, they should be more empowered to contribute to limiting AMR through APM (i.e. screening diagnostics, infection diagnosis, treatment and infection control) in their daily working routines. The observed differences reflect differences in local, national and cross-border structures, and differences in needs of HCW, that need to be considered for safe patient exchange and effective cross-border APM.

**Electronic supplementary material:**

The online version of this article (10.1186/s13756-019-0577-4) contains supplementary material, which is available to authorized users.

## Background

Avoiding antimicrobial resistance (AMR) as well as limiting the spread of multidrug-resistant micro-organisms (MDRO) through AMR prevention measures (APM) is essential for the quality, safety and durability of healthcare and societal health [1, 2]. Core APM are described by various international and national healthcare authorities, and comprise of both timely and adequate screening diagnostics, infection diagnosis, antibiotic treatment, and infection control measures [3–15].

National borders are no barrier for the spread of MDRO. Since the 2011 EU directive on the application of patients’ rights in cross-border healthcare, cross-border mobility of both patient and healthcare workers (HCW) between Germany (DE) and The Netherlands (NL) has steadily increased [16–19]. As a result of the increased cross-border patient and HCW mobility, MDRO may also spread in cross-border regions, like the EUREGIO (i.e. comprising communities of north-eastern NL and north-western DE) [20, 21]. The INTERREG V-A funded initiative EurHealth-1-Health (EH1H) combines the focus on AMR and healthcare through close cross-border cooperation [22]. Close cross-border cooperation was established in particular to address comparisons of APM implemented in both countries, understand differences and find solutions for regional infection control [20].

Previous studies performed within the EUREGIO have focused on differences in the organization of healthcare (e.g. relatively more beds available [23], longer average length of stay [24] and increased connectivity of a higher number of healthcare facilities [20] in DE compared to NL), which are known risk-factors for (the spread of) infections and, thus, indirectly for the spread of AMR [25]. Other studies showed differences in prevalence of MDRO (e.g. lower MRSA admission prevalence in NL) [26–28], and antibiotic prescriptions among outpatients (higher prescription prevalence in Germany) [29]. Differences in AMR and APM between both countries are shaped by a complex combination of interrelated factors [20]. These factors range from differences in regulations [30, 31] to differences in the categorization and designation of MDRO and the recommendations for diagnostic procedures [32–34].

Nonetheless, merely focusing on organisational, regulatory, and procedural factors underestimates one of the most important aspects of successful APM, namely people and particularly HCW [35–38]. HCW are the ones active on the work floor, diagnosing and treating patients, and are thereby largely influencing the success of APM [38–42]. Furthermore, unequivocal and clear communication between HCW is a crucial factor for effective (cross-border) APM [33, 43, 44]. Studying AMR from the HCW’ perspective on both sides of the border will help to develop more effective APM cooperation, because it creates understanding of how HCW perceive the AMR problem and how empowered they feel to tackle the problem through their daily work routines.

This study consisted of a cross-border survey on Dutch and German HCW employed in hospitals of the EUREGIO. The aim of this article is to gain an understanding of the similarities and differences of AMR- and APM-perceptions of Dutch and German HCW that need to be considered for effective cross-border AMR cooperation.

## Methods

In this cross-sectional study, a survey was conducted amongst HCW in hospitals in DE and NL, using an online self-administered questionnaire between June 2017 and July 2018. The bi-national research team consisted of researchers from various specialties, including health sciences, psychology, medical microbiology and epidemiology, infectious diseases, and infection control (see authors). The study was ethically approved by the ethical committee of the University of Twente (BCE18321).

### Setting and participants

The study was performed in six hospitals, which were purposively sampled based on their location in north-eastern NL and north-western DE. The heterogeneous sample consisted of one large university hospital on each side of the border (DE: + − 1500 and NL: + − 1300 beds), as well as one smaller Dutch general hospital (+ − 700 beds) and three smaller German university hospitals (+ − 400–800 beds). Microbiological diagnostics was locally organised in all except one German hospital. In all participating hospitals, local guidelines on antibiotic prescribing were available in the form of (online) formularies. Expert consultations on medical microbiology, infectious diseases and hygiene were available by phone or in person. Because HCW are mainly responsible to perform APM, they were selected as the key-stakeholders. HCW consisted of physicians and nurses of relevant AMR departments (e.g. not psychiatry).

### Survey and distribution

After demographic questions, the survey addressed a variety of AMR-topics, mostly based on a valid and reliable AMR questionnaire [38]. First, questions about the perceived urgency of the AMR problem on various levels, the perceived causes of AMR, beliefs about antibiotic use and the perceived influence that respondents have to limit the AMR problem were asked (1: Fully disagree – 5: Fully agree). Then, we asked questions about APM, which were based on recommendations about APM from various national and international health authorities [3–15] and a study of Dik et al. [45].

APM were introduced to respondents as follows:

- Screening diagnostics: the process of finding out if a patient carries resistant bacteria (incl. asking questions about risk factors for MDRO at admission, taking cultures and testing cultures).
- Infection diagnosis: the diagnosis of an infection (present/absent).
- Treatment: the choice of antibiotics that meets both the patient’s diagnosis and the local antibiotic guidelines.
- Infection control: the implementation of suitable hygiene measures for infection and transmission prevention (e.g. antisepsis, hand hygiene, use of personal protective equipment, and cleaning of equipment and rooms).

The perceived importance of APM was questioned with one item. The perceived influence and perceived availability of resources, knowledge, and social support of colleagues and supervisor on APM was questioned with five items. The perceived influence and perceived availability of resources, knowledge, and social support of colleagues and supervisor were later combined for interpretation into a scale of “feeling sufficiently equipped” for the specific APM.

The survey was originally designed in Dutch for the regional hospital, which was used as a pilot-test for the survey. Tests that were held with a nurse and physician to ensure comprehension and clarity of the questions resulted in small adaptions in wording. After translation by an official translation service to German, the German research team members adapted wordings to better fit the clinical context and jargon. The full survey can be found in Additional file 1.

The survey was developed and administered in Qualtrics, and consisted of 5-point Likert items (Not important–Important, Insufficient–Sufficient). Respondents were informed of the voluntary nature of their participation and confidentiality was guaranteed.

The survey was distributed by email or personal communication followed by snowball sampling with local differences due to practical matters (e.g. local restrictions of using mailing lists and managerial objections with surveys to avoid overload of work for HCW). Reminders were sent twice, but could not be tailored to non-responders.

### Statistical analysis

Descriptive analyses were performed in SPSS (v24). As physicians and nurses have different responsibilities related to AMR [46], results are shown separate per function group. Chi-square tests of homogeneity and Fisher’s exact tests were used to study demographical differences between groups ((i) German and Dutch respondents, (ii) German and Dutch physicians, and (iii) German and Dutch nurses). Mann-Whitney U-tests were used to study differences on the 5-point Likert items between the before mentioned groups. This nonparametric test suits the non-normal distribution of the data, and the nominal nature of the independent variable (i.e. DE/NL) and ordinal nature of the dependent variable (i.e. 5-point Likert items) [47]. Reported *p*-values for the Mann-Whitney U tests are two-tailed (asymptotic-derived p-values presented) and a p-value < 0.05 was considered significant. Possible influence of demographic differences between the German and Dutch groups were considered by comparing Mann-Whitney U tests results with results of Analyses of Covariance (ANCOVA) on ranked responses for each item and scale with age, gender and years of hospital experience as covariates.

## Results

### Respondents

Respondent characteristics are presented in Table 1. Of the 574 respondents, 53% worked in German and 47% worked in Dutch hospitals. German and Dutch respondents differed significantly on all demographic variables included (*p* ≤ 0.001). German physicians were significantly younger (p ≤ 0.001). Dutch nurses were significantly more often female (p ≤ 0.001), were significantly older (*p* = 0.002), and had significantly more experience in the current hospital (*p* = 0.005). Completing the survey took respondents 16 min on average. The respondents of the two hospitals with the highest number of responses represented response rates of less than 19%.

**Table 1:** Survey respondents’ characteristics

Variable & levels		Total	All respondents		Diff. DE/NL	Physicians		Diff. P DE/NL	Nurses		Diff. N DE/NL
		n (%)	DE n (%)	NL n (%)	Test (*P*-value)	DE n (%)	NL n (%)	Test (*P*-value)	DE n (%)	NL n (%)	Test (*P*-value)
#	n (%)	574 (100)	305 (53)	269 (47)	–	128 (22)	49 (9)	–	177 (31)	220 (38)	–
Sex	Male	181 (32)	131 (43)	50 (19)	Chi^2^ (≤0.001)	86 (67)	32 (65)	Chi^2^ (0.812)	45 (25)	18 (8)	Chi^2^ (≤0.001)
	Female	393 (68)	174 (57)	219 (81)		42 (33)	17 (35)		132 (75)	202 (92)	
Age	< 25 years	30 (5)	20 (7)	11 (4)	Chi^2^ (≤0.001)	0 (0)	0 (0)	Fisher’s exact (≤0.001)	20 (11)	10 (5)	Chi^2^ (0.002)
	25–35 years	182 (32)	121 (40)	61 (23)		58 (45)	8 (16)		63 (36)	53 (24)	
	36–45 years	157 (27)	78 (26)	78 (29)		45 (35)	19 (39)		33 (19)	60 (27)	
	46–55 years	129 (22)	60 (20)	67 (25)		17 (13)	10 (20)		43 (24)	59 (27)	
	56–65 years	74 (13)	25 (8)	51 (19)		7 (5)	11 (22)		18 (10)	38 (17)	
	> 65 years	2 (0)	1 (0)	1 (0)		1 (1)	1 (2)		0 (0)	0 (0)	
Hospital	1 (general)	223 (39)	–	223 (83)	–	–	41 (84)	–	–	182 (83)	–
	2 (academic)	252 (44)	251 (82)	–		96 (75)	–		156 (88)	–	
	3 (academic)	46 (8)	–	46 (17)		–	8 (16)		–	38 (17)	
	4 (university)	23 (4)	23 (8)	–		11 (9)	–		12 (7)	–	
	5 (university)	13 (2)	14 (5)	–		9 (7)	–		4 (2)	–	
	6 (university)	12 (2)	12 (4)	–		8 (6)	–		4 (2)	–	
	Other^b^	5 (1)	5 (2)	–		4 (3)	–		1 (1)	–	
Departments^a^	Anaesthesiology	80 (11)	72 (17)	8 (3)	–	34 (19)	5 (10)	–	38 (16)	3 (1)	–
	Intensive Care	79 (11)	63 (15)	17 (6)		25 (14)	4 (2)		38 (16)	13 (5)	
	Paediatrics	77 (11)	42 (10)	35 (11)		14 (8)	1 (1)		28 (12)	34 (13)	
	Surgery	72 (10)	25 (6)	47 (15)		8 (4)	3 (2)		17 (7)	44 (17)	
	Obstetrics/Gynaecology	44 (6)	11 (3)	34 (11)		4 (2)	5 (3)		7 (3)	29 (11)	
	Internal medicine	36 (5)	20 (5)	16 (5)		15 (8)	1 (1)		5 (2)	15 (6)	
	Oncology	33 (5)	24 (6)	11 (4)		9 (5)	0 (0)		15 (6)	11 (4)	
	Orthopaedics	33 (5)	17 (4)	17 (6)		8 (4)	7 (4)		9 (4)	10 (4)	
	Emergency Department	30 (4)	17 (4)	13 (4)		9 (5)	3 (2)		8 (3)	10 (4)	
	Other	235 (33)	124 (30)	111 (36)		52 (29)	20 (11)		72 (30)	91 (35)	
Hospital experience	< 1 year	25 (4)	15 (5)	10 (4)	Chi^2^ (≤0.001)	6 (5)	4 (8)	Chi^2^ (0.333)	9 (5)	6 (3)	Chi^2^ (0.005)
	≥1 year, < 5 years	116 (20)	84 (28)	32 (12)		49 (38)	12 (24)		35 (20)	20 (9)	
	5–10 years	132 (23)	73 (24)	60 (22)		35 (27)	15 (31)		38 (21)	44 (20)	
	> 10 years	301 (52)	133 (44)	167 (62)		38 (30)	18 (37)		95 (54)	150 (68)	

Note. Differences between nationalities are calculated with Chi-square tests of homogeneity (Asymptotic Significance (2-sided) shown) or Fisher’s exact tests (Exact Sig. (2-sided) shown)

^a^Only departments with > 30 respondents in total (DE + NL) are shown. Respondents could select multiple departments (23% of the German and 9% of the Dutch HCW indicated to work at various departments)

^b^Snowball-sampling included five respondents from two other hospitals, both located within the EUREGIO

### Survey results

**Fig. 1:**
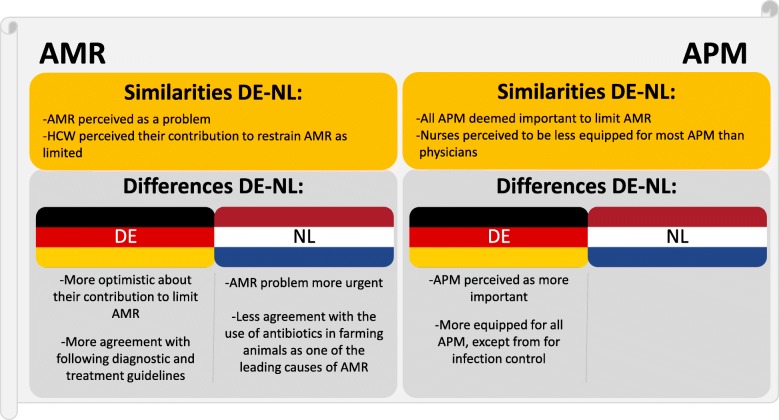
Antimicrobial resistance (AMR) and AMR prevention measures (APM): similarities and differences between German and Dutch respondents

Results of the survey are presented in Table 2 (AMR statements) and 3 (AMR prevention measures). Results compare (i) all respondents (DE-NL), (ii) German physicians and Dutch physicians, and (iii) German nurses and Dutch nurses. Means without standard deviations are merely used as interpretable visualisation of differences between groups (i.e. means closer to one interpreted as disagreement with item and closer to five interpreted as agreement with item) and were not used in any calculations. Full results in the form of percentages per answer category are discussed in text and can be found in, Additional file 2. Similarities and differences of Tables 2 and 3 are summarized in Fig. 1.

**Table 2:** AMR statement responses of (i) all respondents, (ii) German/Dutch physicians, and (iii) German/Dutch nurses, including *p*-values of differences between nationalities

*Statements*		All respondents (n = 574)			Physicians (n = 177)			Nurses (n = 397)		
		DE (n = 305)	NL (n = 269)	P-value	DE (*n* = 128)	NL (*n* = 49)	P-value	DE (n = 177)	NL (*n* = 220)	P-value
		Mean	Mean		Mean	Mean		Mean	Mean	
AMR is a problem for …	the general population.	4.2	**4.6**	**≤0.001**	4.3	**4.5**	**0.026**	4.1	**4.6**	**≤0.001**
	nursing homes.	4.3	4.4	0.968	4.4	4.4	0.851	4.3	4.4	0.859
	our hospital.	4.4	**4.6**	**0.043**	4.3	4.6	0.180	4.4	4.6	0.262
	my patients.	4.2	**4.5**	**0.002**	4.2	4.3	0.281	4.3	**4.5**	**0.017**
One of the leading causes of AMR is …	the use of antibiotics in farming animals.	**4.5**	3.6	**≤0.001**	**4.4**	4.0	**0.004**	**4.5**	3.5	**≤0.001**
	the use of antibiotics by patients.	3.4	**3.6**	**0.011**	3.2	**3.6**	**0.021**	3.5	3.6	0.379
	the admission of nursing home patients.	**2.6**	2.4	**0.006**	2.6	2.5	0.254	**2.6**	2.4	**0.027**
I believe that …	antibiotics are prescribed at the request of patients.	**2.9**	2.4	**≤0.001**	**3.0**	2.4	**0.013**	**2.8**	2.3	**0.001**
	antibiotic prescriptions should be based on lab results.	**4.4**	3.9	**≤0.001**	**4.4**	3.9	**≤0.001**	**4.4**	3.9	**≤0.001**
	I am sufficiently informed about the diagnostic policy.	**3.6**	3.4	**0.002**	3.6	3.8	0.791	**3.6**	3.3	**0.003**
	broad spectrum antibiotics should be provided when there is doubt of an infection.	1.7	**2.2**	**≤0.001**	1.5	**1.5**	**0.001**	1.9	**2.3**	**≤0.001**
	I can contribute sufficiently to limit AMR.	**3.6**	2.8	**≤0.001**	**4.3**	4.3	**≤0.001**	**3.1**	2.6	**≤0.001**

Note. When there is a statistically significant difference between nationalities, the nationality with the highest mean is shown in bold. *DE* Germany, *NL* The Netherlands

**Table 3:** APM responses of (i) all respondents, (ii) German/Dutch physicians, and (iii) German/Dutch nurses, including *p*-values of differences between nationalities

*APM*		All respondents (*n* = 574)			Physicians (*n* = 177)			Nurses (*n* = 397)		
		DE (*n* = 305)	NL (*n* = 269)	*P*-value	DE (*n* = 128)	NL (*n* = 49)	*P*-value	DE (n = 177)	NL (*n* = 220)	*P*-value
		Mean	Mean		Mean	Mean		Mean	Mean	
Screening diagnostics	Importance	**4.7**	4.5	**≤0.001**	**4.7**	4.4	**0.002**	**4.8**	4.6	**≤0.001**
	Feeling sufficiently equipped^a^	**3.6**	3.3	**0.005**	3.7	3.6	0.075	3.5	3.3	0.166
Infection diagnosis	Importance	**4.7**	4.5	**0.003**	**4.7**	4.4	**0.004**	4.6	4.5	0.134
	Feeling sufficiently equipped^a^	**3.6**	3.3	**≤0.001**	4.3	4.2	0.197	3.2	3.1	0.335
Treatment	Importance	**4.8**	4.5	**≤0.001**	**4.9**	4.5	**≤0.001**	**4.8**	4.4	**≤0.001**
	Feeling sufficiently equipped^a^	**3.2**	2.8	**≤0.001**	4.0	4.1	0.746	**2.6**	2.5	**0.004**
Infection control	Importance	**4.8**	4.5	**≤0.001**	**4.7**	4.3	**≤0.001**	**4.9**	4.5	**≤0.001**
	Feeling sufficiently equipped^a^	4.0	3.9	0.230	3.9	3.8	0.534	4.0	3.9	0.114

Note. When there is a statistically significant difference between nationalities, the nationality with the highest mean is shown in bold. DE = Germany, NL = The Netherlands

^a^Cronbach’s alphas for the “Feeling sufficiently equipped”-scales were between 0.69 and 0.83 for all respondents of this study

### AMR problem urgency

Most of the respondents (≥59%) perceived AMR as a problem for the general population, nursing homes, their hospital and their patients. Dutch respondents scored higher than German respondents on statements of AMR being a problem for the general population (*p* ≤ 0.001), their hospital (*p* = 0.043) and their patients (*p* = 0.002), although German respondents also scored relatively high (lowest mean importance score is 4.1). Thus, both German and Dutch respondents perceived AMR as a problem on various levels, and Dutch respondents do so slightly more than German respondents.

### AMR cause

German respondents scored higher than Dutch respondents on statements of the leading causes of AMR being the use of antibiotics in farming animals (*p* ≤ 0.001) and the admission of nursing homes (*p* = 0.006). Dutch respondents scored higher on the statement of the use of antibiotics by patients (*p* = 0.011) as a leading cause of AMR than German respondents.

### Beliefs about antibiotic use

German respondents scored higher on the statement that antibiotics are prescribed at the request of patients (*p* ≤ 0.001) and on statements about antibiotic prescriptions according to guidelines (e.g. antibiotic prescriptions should be based on lab results (p ≤ 0.001), I am sufficiently informed about the diagnostic policy (*p* = 0.002), and broad spectrum antibiotics should *not* be provided when there is doubt of an infection (*p* ≤ 0.001)).

### Contribution to limit AMR

Notably, only 19% of all respondents totally agreed that he/she can sufficiently contribute to limit AMR, and 30% respondents perceive their contribution to limit AMR as insufficient. This is especially true for nurses (lower means than physicians in both countries). German respondents scored higher on the item about being able to sufficiently contribute to limit AMR than their colleagues from The Netherlands (*p* ≤ 0.001). This difference was mainly apparent for nurses, where the means differed more than for physicians (although both reached significance).

### APM importance

All APM were deemed very important to limit AMR by most (≥67%) respondents (see also high importance means). German respondents scored the importance of all APM higher than respondents from The Netherlands, although scores for APM importance were high for both groups (lowest mean importance of Dutch respondents was 4.5).

### Feeling equipped for APM

German respondents scored also higher on the feeling of being equipped at their hospital for specific APM (screening diagnostics *p* = 0.005, infection diagnosis *p* ≤ 0.001, and treatment p ≤ 0.001), although the mean scores did not differ much between German and Dutch respondents.

Both German and Dutch nurses scored feeling sufficiently equipped lower than physicians (lower mean scores) for most APM, although this was not statistically tested. This is less apparent when comparing the means in both groups (physicians-nurses) for infection control.

### Considering demographic differences

The comparison of unadjusted (Mann-Whitney U tests) and adjusted (ranked ANCOVA corrected for age, gender and years of hospital experience) test results can be found in Additional file 3. Of all observed differences that were significant in the unadjusted analyses, only three were not significant in the adjusted analyses (1. all respondents: AMR is a problem in our hospital, 2. physicians: AMR is a problem for the general population, and 3. nurses: one of the leading causes of AMR is the use of antibiotics by patients).

## Discussion

This study aimed to compare German and Dutch HCW in their perceptions of AMR and prevention measures. This was done in order to create understanding of the problem urgency and to learn how HCW perceive their potential contribution to tackle the AMR problem through daily work routines. Understanding and comparing HCW’ perspectives on AMR and APM between countries where patient and HCW mobility is promoted, is essential for safe patient and HCW exchange, and effective cross-border cooperation.

### Differences in HCW’ perspectives on AMR and APM

Especially Dutch nurses felt less able to contribute sufficiently to limit AMR, as reflected in their lower mean score. The resistance rates of several MDRO are higher in German hospitals than in Dutch hospitals (e.g. proportion of MRSA/*S. aureus* from cases of bacteraemia: DE: 9.1% vs. NL: 1,5% and VRE/*E. faecium*: DE: 16.5% vs. NL: 1,4%) [28]. These low MDRO rates are likely a result of the consistent MRSA ‘search and destroy’ policy that The Netherlands implemented early and retained since decades [15, 48], while Germany has shown decreasing incidence rates for MRSA over the past few years by a ‘search and follow’ strategy [49]. Dutch HCW are likely more aware of the urgency of the AMR problem, because of the longstanding search and destroy policy. At the same time, German HCW might be more optimistic about their possible contribution to limit AMR, because they handle MDRO more often in daily practice and – starting from a higher level – the incidence can be decreased more in Germany. Additionally, this powerless feeling might be attributable to the fact that, in the Netherlands more than in Germany, AMR problems at least partially also occur outside of the hospital (e.g. MDRO acquired through traveling, food chains and animals). This is also represented in the differing answers on leading causes of AMR [50–55]. Thus, differences between German and Dutch HCW’ perceptions of the AMR problem urgency and potential contributions might be attributable to differences between both countries in MDRO hospital incidence and APM strategies.

### AMR awareness

As the awareness in both Dutch and German HCW in this study is considerably higher compared to similar studies [40, 56], and because the ongoing EH1H network project and preceding networks (MRSA-net and Eursafety network) in this area already contribute to improving awareness [20, 26], recent and future cross-border AMR prevention strategies in this region do not primarily need to target problem awareness to such an extent as is often suggested for AMR prevention strategies [4]. However, continuing current efforts to retain awareness of the AMR problem in- and outside of hospitals (e.g. the German DART 2020 strategy and the European Antibiotic Awareness Day (EAAD)) [57, 58] is recommended, since no short-term solutions are expected to be found for the complex AMR problems [2, 25].

### HCW empowerment

Astonishingly, only few HCW from both countries perceived their contribution to limit AMR as sufficient. Although German respondents felt slightly more optimistic about their contribution to limit AMR than their Dutch colleagues, their mean score is far from optimistic (3.5).

Therefore, AMR prevention strategies in both countries should primarily focus on the awareness of how HCW can contribute to preventing the (cross-border) spread of MDRO. Studies have shown that improved APM over time, which can only be realized by empowered individual HCW, have led to a regional/national stabilisation or even reduction of MDRO prevalence [26, 59, 60].

Special attention is required for empowering nurses in APM, since nurses are less confident about their role in diagnostics, diagnosis and treatment, as also reflected in this study’s results [46, 61–64]. Nurses are the “eyes and ears” most frequently being in contact with the patient, and can thereby fastest recognize inadequate or suboptimal APM [61, 63, 64]. Empowering HCW starts with promoting pro-active roles of all HCW in all APM components [63]. To empower HCW and specifically nurses, more coordinated and innovative (e.g. problem-based learning) approaches to AMR education and communication are needed, dovetailed to the HCW needs [65–67]. Furthermore, awareness of HCW’ potential contribution to limit AMR can be improved by measuring and reporting APM performance and AMR outcomes data, according to general audit and feedback principles of quality management [68]. Current surveillance efforts in both countries (i.e. PREZIES and KISS [69]) are the basis for reporting such data. Although outcomes (e.g. decreased resistance or less infections) are not easily linked to individual APM actions, incorporating measurements on APM performance and outcome data over the long-term in cyclic learning processes, has shown to improve HCW’ APM performance [59, 69–71].

### Cross-border AMR cooperation

Germany and The Netherlands both have very developed healthcare systems, but the two systems differ considerably from one another in organisational, regulatory and financial structures [72, 73]. Previous studies found that cross-border healthcare is not yet optimal according to HCW, mainly because of communication barriers and non-supportive IT [74–76]. Suboptimal and/or ambiguous communicational and non-supportive IT are known barriers within institutions [46, 77], and will become even more problematic on a national or cross-border level, because of differences in language, taxonomy, and interoperability of IT.

Furthermore, AMR outcomes and APM cooperation in a cross-border setting are not only influenced by HCW’ perceptions and actions, but also by the complex interplay of organisational, regulatory and financial structures that shape a healthcare system [20]. These structures are robust, and dealing with them may be done differently on the level of federal states (“Bundesländer”, DE) and provinces (NL), healthcare institutions and individual HCW. Because of these differences on various levels within both countries, it is difficult to synchronize healthcare systems for cross-border cooperation. Comprehending similarities and differences in healthcare systems and HCW’ perspectives in a cross-border region is an essential step towards successful cross-border APM cooperation.

eHealth has the potential to support and improve synchronisation AMR education, communication, and surveillance and performance feedback in a cross-border region, as has been successfully shown before in AMR studies [45, 78–81]. By following a participatory, holistic and human centred approach for eHealth development and implementation, eHealth has the potential advantage of being able to adapt to differences in the users’ needs (e.g. nurse specific needs) and contexts (e.g. national APM strategies), which is relevant for AMR-cooperation in a cross-border setting. To fully understand the users’ needs and contexts, current initiatives that compare AMR and APM from different perspectives should be continued. Thereby, knowledge and insights from best practices can be exchanged, and innovative eHealth approaches can be developed that ensure the fit between the technology, the users and the cross-border context [82].

### Limitations

This study used a purposive sample of hospitals in the EUREGIO and thus might not represent other cross-border regions, since every cross-border region has its own healthcare system structure and dynamics and its own AMR biotope [17, 83].

Response rates were low, even for the two hospitals that provided the most responses (≤19%). This is most likely attributable to the fact that AMR and APM are not HCW’ core business. Therefore, only HCW with an interest in AMR/APM might have participated (i.e. selection bias), which might have influenced the results to be more positive than they actually are. HCW that do not have that much AMR/APM experience will likely answer more negatively on questions such as feeling sufficiently equipped (see e.g. Björkman et al., 2010 [41]). This would mean that our suggested improvements, such as empowering all HCW in APM, are in reality even more needed to limit the AMR problem.

Furthermore, German and Dutch respondents varied significantly on nearly all demographic characteristics. However, the analyses adjusted for age, sex and years of hospital experience showed that only for a small number of questions the observed differences in HCW’ perspectives could be (partially) explained by demographic differences.

Other limitations relate to the use of Likert items. Central tendency bias might have occurred by respondents avoiding choosing the extreme response categories (scores 1 & 5) [84]. We do not see this bias reflected in the answers, since respondents scored extreme responses on questions where we expected mostly positive (e.g. importance of AMR prevention measures) or mostly negative (e.g. broad-spectrum antibiotics should be provided when there is doubt of an infection) answers. Social desirability bias might always have occurred, since most people are aware that AMR should require special attention (note that this does not mean that it in daily working routines) [84].

The survey used was based on a validated questionnaire, used elements from health authorities’ recommendations on APM [1, 3–8, 38], and was discussed with experts in the field of AMR, but was not validated itself. To be able to use this survey as a tool to compare HCW’ perspectives between countries or even evaluate intervention effects, it should be further tested elaborately and validated [84] (see for example Teixeira Rodrigues, et al. [38]).

Despite these limitations, we do believe that this survey proved useful for a primary identification of HCW’ perspectives. This study can be seen as an essential step towards safer patient exchange and improved cross-border cooperation, since the cross-border AMR problem has, to our best knowledge, not been studied before from the HCW’ perspective.

## Conclusion

Both German and Dutch HCW are aware of the AMR problem, but both perceive their influence to limit AMR as insufficient. HCW do acknowledge the importance of APM (i.e. screening diagnostics, infection diagnosis, treatment and infection control) they perform in their daily working routines to limit AMR, but do not feel sufficiently equipped to do so. Therefore, AMR strategies should not primarily focus on emphasizing the relevance of APM, but should rather focus on empowering HCW in their working routines by providing them with the tools, knowledge and skills they need to limit AMR.

Because of robust national healthcare structures, adaptive solutions are essential to tackle the challenges caused by AMR on a regional level. APM should be tailored to work in regional or even local settings, and need to be implemented by committed HCW. Thus, developing and implementing (cross-border) APM requires a comprehensive understanding of the contexts in which they will be implemented and the people that will execute the strategies (i.e. HCW). The similarities and differences between German and Dutch HCW as found in this study, can serve as a primary identification of factors that need to be considered for cross-border APM cooperation.

## Additional files

Additional file 1: Survey on AMR and APM. AMR/APM survey (DOCX 15 kb)

Additional file 2: **Table S2**a. Statement responses of (i) all respondents, (ii) German/Dutch physicians, and (iii) German/Dutch nurses, including *p*-values of differences between nationalities. **Table S3**a. APM responses of (i) all respondents, (ii) German/Dutch physicians, and (iii) German/Dutch nurses, including *p*-values of differences between nationalities. Full results on AMR (2a) and APM (3a) in the form of percentages per answer category. (DOCX 33 kb)

Additional file 3: **Table S2**b. Ranked ANCOVA (corrected for age, gender and years of hospital experience) results of (i) all respondents, (ii) German/Dutch physicians, and (iii) German/Dutch nurses, including *p*-values of differences between nationalities. **Table S3**b. Ranked ANCOVA (corrected for age, gender and years of hospital experience) results of (i) all respondents, (ii) German/Dutch physicians, and (iii) German/Dutch nurses, including p-values of differences between nationalities. Comparison of unadjusted (Mann-Whitney U tests) adjusted (ranked ANCOVA (corrected for age, gender and years of hospital experience)) results. (DOCX 32 kb)

## Data Availability

The datasets generated and/or analysed during the current study are not publicly available due to privacy restrictions, but are available from the corresponding author on reasonable request.

## Abbreviations

AMR: Antimicrobial resistance
APM: Antimicrobial resistance prevention measures
DE: Germany
EH1H: EurHealth-1-Health
HCW: Healthcare workers
KRINKO: Commission for Hospital Hygiene and Infection Prevention (DE: Kommission für Krankenhaushygiene und Infektionsprävention)
MDRO: Multidrug-resistant micro-organisms
MRSA: Methicillin-resistant *Staphylococcus aureus*
NL: The Netherlands

## References

[1] World Health Organization [WHO] (2012). The evolving threat of antimicrobial resistance: options for action.

[2] O'Neill J (2014). Antimicrobial resistance: tackling a crisis for the health and wealth of nations.

[3] Storr J (2017). Core components for effective infection prevention and control programmes: new WHO evidence-based recommendations. Antimicrob Resist Infect Control.

[4] World Health Organization [WHO] (2015). Global action plan on antimicrobial resistance.

[5] World Health Organization [WHO] (2016). Diagnostic stewardship: a guide to implementation in antimicrobial resistance surveillance sites.

[6] Tacconelli E (2014). ESCMID guidelines for the management of the infection controlmeasures to reduce transmission of multidrug-resistant gram-negative bacteria in hospitalized patients. Clin Microbiol Infect.

[7] National Quality Forum (2016). National Quality Partners Playbook: antibiotic stewardship in acute care.

[8] European Commission. A European one health action plan against antimicrobial resistance (AMR). Brussels: European Commission; 2017.

[9] Kommission für Krankenhaushygiene und Infektionsprävention [KRINKO] beim Robert Koch-Institut [RKI] (2014). Empfehlungen zur Prävention und Kontrolle von methicillin-resistenten Staphylococcus aureus-Stämmen (MRSA) in medizinischen und pflegerischen Einrichtungen. Bundesgesundheitsbl Gesundheitsforsch Gesundheitsschutz.

[10] Kommission für Krankenhaushygiene und Infektionsprävention [KRINKO] beim Robert Koch-Institut [RKI] (2018). Hygienemaßnahmen zur Prävention der Infektion durch Enterokokken mit speziellen Antibiotikaresistenzen. Bundesgesundheitsbl Gesundheitsforsch Gesundheitsschutz.

[11] Kommission für Krankenhaushygiene und Infektionsprävention [KRINKO] beim Robert Koch-Institut [RKI] (2012). Hygienemaßnahmen bei Infektionen oder Besiedlung mit multiresistenten gramnegativen Stäbchen. Bundesgesundheitsbl Gesundheitsforsch Gesundheitsschutz.

[12] Stichting Werkgroep AntibioticaBeleid [SWAB] (2012). Herziening SWAB richtlijn Behandeling MRSA dragers.

[13] Stichting Werkgroep AntibioticaBeleid [SWAB] (2017). SWAB guidelines for antimicrobial stewardship.

[14] Stichting Werkgroep AntibioticaBeleid [SWAB] (2018). SWAB richtlijn: selectieve decontaminatie bij patiënten op de intensive care.

[15] Werkgroep Infectie Preventie [WIP] van het Rijksinstituut voor Volksgezondheid en Milieu [RIVM] (2018). WIP Richtlijnen Ziekenhuizen [ZKH].

[16] Parliament., E (2011). The application of patients’ rights in cross-border healthcare.

[17] Glinos IA, Baeten R (2015). Reprint of: dream vs. reality: seven case-studies on the desirability and feasibility of cross-border hospital collaboration in Europe. Soc Sci Med.

[18] Jutten K, Janssens P. Patiënten zonder grenzen: grensoverschrijdende patiëntenstromen in de benelux. Brussels: Benelux Unie; 2016.

[19] Costigliola V (2011). Mobility of medical doctors in cross-border healthcare. EPMA J.

[20] Ciccolini M (2013). Infection prevention in a connected world: the case for a regional approach. Int J Med Microbiol.

[21] Donker T (2012). Hospital networks and the dispersal of hospital-acquired pathogens by patient transfer. PLoS One.

[22] EurHealth-1-Health (2018). Project | EurHealth-1Health.

[23] Eurostat (2016). Health care.

[24] Eurostat (2016). In-patient average length of stay (days).

[25] Holmes AH (2016). Understanding the mechanisms and drivers of antimicrobial resistance. Lancet.

[26] Friedrich AW (2008). EUREGIO MRSA-net Twente/Münsterland – a Dutch-German cross-border network for the prevention and control of infections caused by methicillin-resistant Staphylococcus aureus. Eurosurveillance.

[27] Köck R (2009). Cross-border comparison of the admission prevalence and clonal structure of meticillin-resistant Staphylococcus aureus. J Hosp Infect.

[28] EARS-net. *ECDC surveillance atlas of infectious diseases*. In: *EARS-net. *Stockholm: ECDC; 2017.

[29] Dik JH (2016). Cross-border comparison of antibiotic prescriptions among children and adolescents between the north of the Netherlands and the north-west of Germany. Antimicrob Resist Infect Control.

[30] Robert Koch Institut. RKI. Commission for Hospital Hygiene and Infection Prevention [KRINKO]; 2016. [cited 2019 21–2]; Available from: https://www.rki.de/EN/Content/Institute/Committees/KRINKO/KRINKO_node_en.html.

[31] Rijksinstituut voor Volksgezondheid en Milieu [RIVM]. Werkgroep Infectie Preventie [WIP]; 2019. [cited 2019 21–2]; Available from: https://www.rivm.nl/werkgroep-infectie-preventie-wip.

[32] Müller J (2015). Cross-border comparison of the Dutch and German guidelines on multidrug-resistant gram-negative microorganisms. Antimicrob Resist Infect Control.

[33] Köck Robin, Siemer Philipp, Esser Jutta, Kampmeier Stefanie, Berends Matthijs, Glasner Corinna, Arends Jan, Becker Karsten, Friedrich Alexander (2018). Defining Multidrug Resistance of Gram-Negative Bacteria in the Dutch–German Border Region—Impact of National Guidelines. Microorganisms.

[34] Podbielski A (2018). MiQ: Qualitätsstandards in der mikrobiologisch-infektiologischen Diagnostik.

[35] Hulscher MEJL, Grol RPTM, van der Meer JWM (2010). Antibiotic prescribing in hospitals: a social and behavioural scientific approach. Lancet Infect Dis.

[36] Schuts EC (2016). Current evidence on hospital antimicrobial stewardship objectives: a systematic review and meta-analysis. Lancet Infect Dis.

[37] McCullough AR (2015). Not in my backyard: a systematic review of clinicians' knowledge and beliefs about antibiotic resistance. J Antimicrob Chemother.

[38] Teixeira Rodrigues A (2016). Physicians' attitudes and knowledge concerning antibiotic prescription and resistance: questionnaire development and reliability. BMC Infect Dis.

[39] Gonzalez-Gonzalez C (2015). Effect of Physicians' attitudes and knowledge on the quality of antibiotic prescription: a cohort study. PLoS One.

[40] Pulcini C (2017). Junior doctors’ knowledge and perceptions of antibiotic resistance and prescribing: a survey in France and Scotland. Clin Microbiol Infect.

[41] Bjorkman I (2010). Perceptions among Swedish hospital physicians on prescribing of antibiotics and antibiotic resistance. Qual Saf Health Care.

[42] Easton PM (2007). Infection control and management of MRSA: assessing the knowledge of staff in an acute hospital setting. J Hosp Infect.

[43] Birgand G (2015). Overcoming the obstacles of implementing infection prevention and control guidelines. Clin Microbiol Infect.

[44] Edwards R (2012). Communication strategies in acute health care: evaluation within the context of infection prevention and control. J Hosp Infect.

[45] Dik JH (2015). An integrated stewardship model: antimicrobial, infection prevention and diagnostic (AID). Future Microbiol.

[46] Keizer J (2018). *EurHealth-1-health: supporting healthcare workers to limit antibiotic resistance in hospitals*. *Supporting Health by Technology VIII* 2018.

[47] Harpe SE (2015). How to analyze Likert and other rating scale data. Curr Pharm Teach Learn.

[48] Wertheim HF (2004). Low prevalence of methicillin-resistant Staphylococcus aureus (MRSA) at hospital admission in the Netherlands: the value of search and destroy and restrictive antibiotic use. J Hosp Infect.

[49] Jurke A, Köck R, Becker K, Thole S, Hendrix R, Rossen J, Daniels-Haardt I, Friedrich A (2013). Molecular epidemiology of meticillin-resistant Staphylococcus aureus (MRSA): think regionally but use globally uniform typing languages. Eurosurveillance.

[50] Köck R (2016). Persistence of nasal colonization with human pathogenic bacteria and associated antimicrobial resistance in the German general population. New Microbes New Infect..

[51] Meyer E (2012). Pet animals and foreign travel are risk factors for colonisation with extended-spectrum beta-lactamase-producing Escherichia coli. Infection.

[52] Overdevest I (2011). Extended-spectrum beta-lactamase genes of Escherichia coli in chicken meat and humans*, The Netherlands*. Emerg Infect Dis.

[53] Tangden T (2010). Foreign travel is a major risk factor for colonization with Escherichia coli producing CTX-M-type extended-spectrum beta-lactamases: a prospective study with Swedish volunteers. Antimicrob Agents Chemother.

[54] Schmithausen RM (2015). Analysis of transmission of MRSA and ESBL-E among pigs and farm personnel. PLoS One.

[55] Murray CK, Blyth DM (2017). Acquisition of Multidrug-Resistant Gram-Negative Organisms during travel. Mil Med.

[56] Guerra CM (2007). Physicians’ perceptions, beliefs, attitudes, and knowledge concerning antimicrobial resistance in a Brazilian teaching hospital. Infect Control Hosp Epidemiol.

[57] Bundesministerium für Gesundheit. DART 2020 - deutsche Antibiotika-Resistenzstrategie; 2019. [cited 2019 21–2]; Available from: https://www.bundesgesundheitsministerium.de/themen/praevention/antibiotika-resistenzen/antibiotika-resistenzstrategie.html.

[58] European Centre for Disease Prevention and Control [ECDC] of the European Union [EU]. *Key messages for professionals in hospitals and other healthcare settings*. European Antibiotic Awareness Day 2019 [cited 2019 02–26]; Available from: http://antibiotic.ecdc.europa.eu/en/get-informed/key-messages/key-messages-professionals-hospitals-and-other-healthcare-settings.

[59] Dyar OJ (2017). Managing responsible antimicrobial use: perspectives across the healthcare system. Clin Microbiol Infect.

[60] Vos MC (2009). 5 years of experience implementing a methicillin-resistant Staphylococcus aureus search and destroy policy at the Largest University medical Center in the Netherlands. Infect Control Hosp Epidemiol.

[61] Wentzel J (2016). Antibiotic information application offers nurses quick support. Am J Infect Control.

[62] Verhoeven F, Verhoeven F (2009). When staff handle staph.

[63] Charani E, Holmes AH (2013). Antimicrobial stewardship programmes: the need for wider engagement. BMJ Qual Saf.

[64] Broom A (2017). Nurses as antibiotic brokers: institutionalized praxis in the hospital. Qual Health Res.

[65] Gholami M (2016). Comparing the effects of problem-based learning and the traditional lecture method on critical thinking skills and metacognitive awareness in nursing students in a critical care nursing course. Nurse Educ Today.

[66] Rogers Van Katwyk S, Jones SL, Hoffman SJ (2018). Mapping educational opportunities for healthcare workers on antimicrobial resistance and stewardship around the world. Hum Resour Health.

[67] Hofstede G (2019). Cultures and organizations: software of the mind | SpringerLink.

[68] Ivers N, et al. Audit and feedback: effects on professional practice and healthcare outcomes. Cochrane Database Syst Rev. 2012;(6):CD000259. 10.1002/14651858.CD000259.pub3 PMC11338587 22696318

[69] Manniën J (2007). Comparison of the National Surgical Site Infection surveillance data between the Netherlands and Germany: PREZIES versus KISS. J Hosp Infect.

[70] Freeman R (2013). Advances in electronic surveillance for healthcare-associated infections in the 21st century: a systematic review. J Hosp Infect.

[71] Baysari Melissa T., Lehnbom Elin C., Li Ling, Hargreaves Andrew, Day Richard O., Westbrook Johanna I. (2016). The effectiveness of information technology to improve antimicrobial prescribing in hospitals: A systematic review and meta-analysis. International Journal of Medical Informatics.

[72] Björnberg A, Phang AY, H.C. Powerhouse (2019). Euro health consumer index 2018. *Euro health consumer index*.

[73] Organisation for Economic Co-operation and Development [OECD] of the European Union [EU]. Health at a glance: Europe 2018: State of Health in the EU Cycle. Paris: OECD; 2018.

[74] Glonti K (2015). European health professionals’ experience of cross-border care through the lens of three common conditions. Eur J Intern Med.

[75] Verra SE, Kroeze R, Ruggeri K (2016). Facilitating safe and successful cross-border healthcare in the European Union. Health Policy.

[76] Ferlie E, Ferlie E (2013). The limited role of information and communication technologies in managed networks. Making wicked problems governable?: the case of managed networks in health care.

[77] Keller SC (2018). Ambulatory antibiotic stewardship through a human factors engineering approach: a systematic review. J Am Board Fam Med.

[78] Beerlage-de Jong N (2017). Technology to support integrated antimicrobial stewardship programs: a user centered and stakeholder driven development approach. Infect Dis Rep.

[79] Wentzel J (2014). Participatory eHealth development to support nurses in antimicrobial stewardship. BMC Med Inform Decis Mak.

[80] Beerlage-de Jong N (2018). Combining user-centered design with the persuasive systems design model; the development process of a web-based registration and monitoring system for healthcare-associated infections in nursing homes. Int J Adv Life Sci.

[81] Micallef C (2018). The secondary use of data from hospital electronic prescribing and pharmacy systems to support the quality and safety of antimicorbial use: a systematic review. J Antimicrob Chemother.

[82] Van Gemert-Pijnen JEWC (2018). eHealth research, theory and development: a multidisciplinary approach.

[83] Schmithausen Ricarda, Schulze-Geisthoevel Sophia, Heinemann Céline, Bierbaum Gabriele, Exner Martin, Petersen Brigitte, Steinhoff-Wagner Julia (2018). Reservoirs and Transmission Pathways of Resistant Indicator Bacteria in the Biotope Pig Stable and along the Food Chain: A Review from a One Health Perspective. Sustainability.

[84] Rossi PH, Wright JD, Anderson AB. Handbook of Survey Research. Quantitative Studies in Social Relations. Cambridge: Academic Press; 2013.

