# Correction to: Cross-border comparison of antimicrobial resistance (AMR) and AMR prevention measures: the healthcare workers’ perspective

**DOI:** 10.1186/s13756-019-0589-0

**Published:** 2019-08-08

**Authors:** J. Keizer, L. M. A. Braakman-Jansen, S. Kampmeier, R. Köck, N. Al Naiemi, R. Te Riet-Warning, N. Beerlage-De Jong, K. Becker, J. E. W. C. Van Gemert-Pijnen

**Affiliations:** 1 Department of Psychology, Health and Technology, Centre for eHealth and Wellbeing Research, University of Twente, P.O. Box 217, 7500AE Enschede, The Netherlands; 2 Institute of Hygiene, University Hospital Münster, Münster, Germany; 3 Institute of Medical Microbiology, University Hospital Münster, Münster, Germany; 4 Institute of Hospital Hygiene Oldenburg, Oldenburg, Germany; 5 Department of Infection Prevention, Hospital Group Twente, Almelo/Hengelo, Netherlands; 6 LabMicTA, Hengelo, Netherlands

**Correction to: Antimicrob Resist Infect Control (2019) 8:123**

**https://doi.org/10.1186/s13756-019-0577-4**

The original article [1] contained **a** minor editorial typo in the following sentence which has now been corrected:

‘Social desirability bias might always have occurred, since most people are aware that AMR should require special attention (note that this does not mean that it gets special attention in daily working routines).’
