# Supplementary material for: Cross-border comparison of antimicrobial resistance (AMR) and AMR prevention measures: the healthcare workers’ perspective
Source: Antimicrob Resist Infect Control. 2019 Jul 22;8:123. doi: 10.1186/s13756-019-0577-4 (PMC6647090; doi:10.1186/s13756-019-0577-4)
Supplement: Supplementary file 1 — Survey on AMR and APM. AMR/APM survey (DOCX 15 kb) [file 13756_2019_577_MOESM1_ESM.docx]

## Additional file 1.

**Title:**

Survey on AMR and APM

**Description:**

AMR/APM survey

**Survey: Antimicrobial Resistance & Infection Control**

**Combat antibiotic resistance**

The durability of modern healthcare is threatened by antibiotic resistance. The “hospital” and University of Twente (UT) are fighting together against antibiotic resistance within the framework of the European INTERREG project.

This survey is about your experiences with antibiotic resistance and infection prevention in hospitals.

It takes about 15 minutes to complete the questionnaire and the results are processed anonymously. Online version: “weblink“.

**Sociodemographic information**

Q1. What is your age? (Ο <25 years, Ο 25-35 years, Ο 36-45 years, Ο 46-55 years, Ο 56-65 years, Ο >65 years)

Q2. What is your gender? (Ο Female, Ο Male)

Q3. In which hospital do you work? (Ο ”Hospital”, etc.)

Q4. At which department do you work? (Ο ”Anaesthesiology”, etc.)

Q5. What is your function? (Ο Medical specialist, Ο Nurse).

Q6. How many years of experience do you have at this hospital? (Ο <1 year, Ο ≥1 year <5 years, Ο 5-10years, Ο >10 years)

You have indicated that you are a nurse. Perhaps not all questions can be answered based on your primary duties / responsibilities (e.g. prescribing antibiotics), but researchers expect you to have an opinion on these issues. We therefore ask you to answer the questions in the best possible way based on your work experience and your cooperation with doctors.

**Part 1: Your experiences with the antimicrobial resistance problem**

Please indicate on a scale of 1 (Fully disagree) to 5 (Fully agree) to what extent you agree with these statements.

Q1a. AMR is a problem for public health.

Q1b. AMR is a problem for nursing homes.

Q1c. AMR is a problem for our hospital.

Q1d. AMR is a problem for my patients.

Q2a. One of the leading causes of AMR is the improper use of antibiotics in farming animals.

Q2b. One of the leading causes of AMR is the improper use of antibiotics by patients.

Q2c. One of the leading causes of AMR is the transfer of nursing home patients to the hospital.

Q3. I believe that antibiotics are prescribed at the request of patients.

Q4. I believe that antibiotic prescriptions should be based on lab results.

Q5. I am sufficiently informed about the diagnostic policy.

Q5. I believe that broad spectrum antibiotics should be provided when there is doubt of an infection.

Q6. I believe that I can contribute sufficiently to limit AMR.

**Part 2: Your experiences with hospital processes related to antimicrobial resistance**

*Part 2A: Screening diagnostics*

The process of finding out if a patient carries a resistant bacterium (incl. screening, taking cultures and testing cultures).

Q2A.1. How important do you think screening diagnostics are to limit AMR? (1: Not important-5: Very important)

Q2A.2. Do you feel like you have sufficient influence on screening diagnostics to limit AMR? (1: Insufficient-5: Sufficient)

Q2A.3. Do you have sufficient resources for screening diagnostics to limit AMR? (1: Insufficient-5: Sufficient)

Q2A.4. Do you have sufficient knowledge for screening diagnostics to limit AMR? (1: Insufficient-5: Sufficient)

*Part 2B: Infection diagnosis*

The diagnosis of an infection (present/absent).

Q2B.1. How important do you think the infection diagnosis is to limit AMR? (1: Not important-5: Very important)

Q2B.2. Do you feel like you have sufficient influence on the infection diagnosis to limit AMR? (1: Insufficient-5: Sufficient)

Q2B.3. Do you have sufficient resources for the infection diagnosis to limit AMR? (1: Insufficient-5: Sufficient)

Q2B.4. Do you have sufficient knowledge for the infection diagnosis to limit AMR? (1: Insufficient-5: Sufficient)

*Part 2C: Treatment*

The choice of antibiotics that meets both the patient's diagnosis and the local antibiotic guidelines.

Q2C.1. How important do you think the treatment is to limit AMR? (1: Not important-5: Very important)

Q2C.2. Do you feel like you have sufficient influence on the treatment to limit AMR? (1: Insufficient-5: Sufficient)

Q2C.3. Do you have sufficient resources for the treatment to limit AMR? (1: Insufficient-5: Sufficient)

Q2C.4. Do you have sufficient knowledge for the treatment to limit AMR? (1: Insufficient-5: Sufficient)

Q2C.5. Do you have sufficient support from colleagues for the treatment to limit AMR? (1: Insufficient-5: Sufficient)

Q2C.6. Do you have sufficient support from your supervisor for the treatment to limit AMR? (1: Insufficient-5: Sufficient)

*Part 2D: Infection control*

The implementation of suitable hygiene measures for infection and transmission prevention (e.g. antisepsis, hand hygiene, use of personal protective equipment, and cleaning of equipment and rooms).

Q2D.1. How important do you think infection control is to limit AMR? (1: Not important-5:Very important)

Q2D.2. Do you feel like you have sufficient influence on infection control to limit AMR? (1: Insufficient-5: Sufficient)

Q2D.3. Do you have sufficient resources for infection control to limit AMR? (1: Insufficient-5: Sufficient)

Q2D.4. Do you have sufficient knowledge for infection control to limit AMR? (1: Insufficient-5: Sufficient)

Q2D.5. Do you have sufficient support from colleagues for infection control to limit AMR? (1: Insufficient-5: Sufficient)

Q2D.6. Do you have sufficient support from your supervisor for infection control to limit AMR? (1: Insufficient-5: Sufficient)

**End of the survey**

This is the end of the survey. Thank you for participating in this study on behalf of the research team. Would you like to be updated about the results of this study and participate in follow-up studies? Please enter you email-address here.
