# Supplementary material for: Cross-border comparison of antimicrobial resistance (AMR) and AMR prevention measures: the healthcare workers’ perspective
Source: Antimicrob Resist Infect Control. 2019 Jul 22;8:123. doi: 10.1186/s13756-019-0577-4 (PMC6647090; doi:10.1186/s13756-019-0577-4)
Supplement: Supplementary file 2 — Table S2a. Statement responses of (i) all respondents, (ii) German/Dutch physicians, and (iii) German/Dutch nurses, including p-values of differences between nationalities. Table S3a. APM responses of (i) all respondents, (ii) German/Dutch physicians, and (iii) German/Dutch nurses, including p-values of differences between nationalities. Full results on AMR (2a) and APM (3a) in the form of percentages per answer category. (DOCX 33 kb) [file 13756_2019_577_MOESM2_ESM.docx]

# Additional file 2

**Title:**

Table S2a. Statement responses of (i) all respondents, (ii) German/Dutch physicians, and (iii) German/Dutch nurses, including p-values of differences between nationalities.

Table S3a. APM responses of (i) all respondents, (ii) German/Dutch physicians, and (iii) German/Dutch nurses, including p-values of differences between nationalities.

**Description:**

Full results on AMR (2a) and APM (3a) in the form of percentages per answer category.

## Table S2a. Statement responses of (i) all respondents, (ii) German/Dutch physicians, and (iii) German/Dutch nurses, including p-values of differences between nationalities.

| ***Statements*** | | **Differences DE-NL** | | | | | | | | | | | | **Physicians (n=177)** | | | | | | | | | | | **Nurses (n=397)** | | | | | | | | | | | |
| --- | --- | --- | --- | --- | --- | --- | --- | --- | --- | --- | --- | --- | --- | --- | --- | --- | --- | --- | --- | --- | --- | --- | --- | --- | --- | --- | --- | --- | --- | --- | --- | --- | --- | --- | --- | --- |
|  |  | **DE** (n=305) | | | | | **NL** (n=269) | | | | | P -value | **DE** (n=128) | | | | | | **NL** (n=49) | | | | | P-value | **DE** (n=177) | | | | | | **NL** (n=220) | | | | | P-value |
|  |  | 1 | 2 | 3 | 4 | 5 | 1 | 2 | 3 | 4 | 5 |  | 1 | | 2 | 3 | 4 | 5 | 1 | 2 | 3 | 4 | 5 |  | 1 | 2 | 3 | 4 | 5 | 1 | | 2 | 3 | 4 | 5 |  |
| AMR is a problem for… | the general population. | 2 | 5 | 16 | 26 | 50 | 0 | 1 | 7 | 22 | 70 | **≤0.001** | 2 | | 3 | 16 | 28 | 52 | 2 | 4 | 4 | 17 | 73 | **0.018** | 3 | 7 | 16 | 25 | 49 | 0 | | 0 | 8 | 22 | 70 | **≤0.001** |
|  | nursing homes. | 4 | 3 | 10 | 23 | 60 | 1 | 1 | 10 | 31 | 57 | 0.968 | 2 | | 3 | 9 | 26 | 59 | 2 | 0 | 6 | 35 | 56 | 0.096 | 5 | 3 | 10 | 21 | 62 | 0 | | 1 | 10 | 30 | 57 | 0.859 |
|  | our hospital. | 3 | 3 | 10 | 23 | 61 | 0 | 1 | 6 | 25 | 67 | **0.043** | 1 | | 4 | 12 | 26 | 57 | 2 | 0 | 4 | 29 | 65 | 0.224 | 4 | 3 | 7 | 21 | 64 | 0 | | 2 | 6 | 25 | 68 | 0.262 |
|  | my patients. | 3 | 6 | 12 | 24 | 55 | 1 | 4 | 6 | 22 | 67 | **0.002** | 1 | | 7 | 16 | 26 | 51 | 2 | 4 | 15 | 19 | 60 | 0.350 | 4 | 5 | 10 | 23 | 58 | 1 | | 4 | 4 | 23 | 68 | **0.017** |
| One of the leading causes of AMR is… | the use of antibiotics in farming animals. | 3 | 1 | 7 | 22 | 67 | 4 | 7 | 38 | 32 | 19 | **≤0.001** | 1 | | 2 | 11 | 28 | 59 | 4 | 0 | 19 | 42 | 35 | **0.006** | 5 | 1 | 5 | 18 | 72 | 4 | | 8 | 43 | 30 | 15 | **≤0.001** |
|  | the use of antibiotics by patients. | 8 | 11 | 33 | 30 | 17 | 3 | 11 | 29 | 38 | 20 | **0.011** | 11 | | 14 | 36 | 24 | 16 | 4 | 10 | 27 | 35 | 23 | **0.027** | 6 | 10 | 31 | 36 | 18 | 2 | | 11 | 29 | 39 | 19 | 0.379 |
|  | the admission of nursing home patients. | 18 | 24 | 40 | 14 | 4 | 17 | 33 | 43 | 6 | 0 | **0.006** | 15 | | 25 | 45 | 13 | 2 | 8 | 42 | 46 | 2 | 2 | 0.214 | 20 | 23 | 37 | 15 | 6 | 19 | | 32 | 42 | 7 | 0 | **0.027** |
| I believe that… | antibiotics are prescribed at the request of patients. | 24 | 16 | 23 | 24 | 14 | 27 | 35 | 19 | 15 | 4 | **≤0.001** | 21 | | 15 | 24 | 22 | 18 | 23 | 38 | 19 | 15 | 6 | **0.012** | 25 | 16 | 22 | 25 | 11 | 28 | | 35 | 19 | 15 | 4 | **0.001** |
|  | antibiotic prescriptions should be based on lab results. | 1 | 3 | 11 | 24 | 61 | 4 | 9 | 16 | 40 | 32 | **≤0.001** | 0 | | 5 | 12 | 22 | 60 | 0 | 13 | 13 | 48 | 27 | **≤0.001** | 1 | 2 | 11 | 25 | 61 | 5 | | 9 | 16 | 38 | 33 | **≤0.001** |
|  | I am sufficiently informed about the diagnostic policy. | 10 | 13 | 16 | 21 | 39 | 6 | 17 | 26 | 33 | 18 | **0.002** | 11 | | 14 | 16 | 19 | 40 | 2 | 8 | 19 | 50 | 21 | 0.888 | 10 | 13 | 18 | 22 | 37 | 7 | | 19 | 27 | 29 | 18 | **0.003** |
|  | broad spectrum antibiotics should be provided when there is doubt of an infection. | 54 | 30 | 10 | 2 | 4 | 33 | 30 | 23 | 12 | 3 | **≤0.001** | 64 | | 27 | 5 | 1 | 3 | 40 | 38 | 15 | 8 | 0 | **0.002** | 46 | 32 | 15 | 3 | 4 | 31 | | 28 | 25 | 13 | 3 | **≤0.001** |
|  | I can contribute sufficiently to limit AMR. | 7 | 14 | 23 | 27 | 30 | 13 | 27 | 36 | 16 | 7 | **≤0.001** | 2 | | 5 | 9 | 36 | 49 | 0 | 15 | 31 | 42 | 13 | **≤0.001** | 10 | 21 | 33 | 20 | 15 | 16 | | 30 | 37 | 10 | 6 | **≤0.001** |
| Note. All numbers shown are percentages, except from p-values; these are p-values of the Mann-Whitney U-test: Asymptotic Sig. (2-sided test). When there was a statistical differences (based on Mann-Whitney U-test) between nationalities, percentages of the nationality with the highest mean ranks are shown in bold DE=Germany, NL=The Netherlands | | | | | | | | | | | | | | | | | | | | | | | | | | | | | | | | | | | | |

## Table S3a. APM responses of (i) all respondents, (ii) German/Dutch physicians, and (iii) German/Dutch nurses, including p-values of differences between nationalities.

| ***APM*** | | **Differences DE-NL (all respondents)** | | | | | | | | | | P-value | **Physicians (n=177)** | | | | | | | | | | P-value | **Nurses (n=397)** | | | | | | | | | | P-value |
| --- | --- | --- | --- | --- | --- | --- | --- | --- | --- | --- | --- | --- | --- | --- | --- | --- | --- | --- | --- | --- | --- | --- | --- | --- | --- | --- | --- | --- | --- | --- | --- | --- | --- | --- |
|  |  | **DE** (n=305) | | | | | **NL** (n=269) | | | | |  | **DE** (n=128) | | | | | **NL** (n=49) | | | | |  | **DE** (n=177) | | | | | **NL** (n=220) | | | | |  |
|  |  | 1 | 2 | 3 | 4 | 5 | 1 | 2 | 3 | 4 | 5 |  | 1 | 2 | 3 | 4 | 5 | 1 | 2 | 3 | 4 | 5 |  | 1 | 2 | 3 | 4 | 5 | 1 | 2 | 3 | 4 | 5 |  |
| SD | Importance | 0 | 1 | 3 | 18 | 78 | 0 | 1 | 3 | 18 | 78 | **≤0.001** | 0 | 2 | 4 | 22 | 72 | 0 | 4 | 2 | 46 | 48 | **0.004** | 0 | 0 | 3 | 16 | 81 | 0 | 0 | 4 | 32 | 63 | **≤0.001** |
|  | Influence | 13 | 20 | 22 | 25 | 20 | 16 | 16 | 26 | 27 | 16 | 0.489 | 8 | 14 | 24 | 29 | 26 | 8 | 13 | 25 | 35 | 19 | 0.708 | 16 | 25 | 21 | 21 | 16 | 17 | 16 | 26 | 25 | 15 | 0.518 |
|  | Resources | 5 | 7 | 21 | 26 | 41 | 5 | 9 | 31 | 30 | 25 | **≤0.001** | 2 | 5 | 21 | 27 | 44 | 2 | 8 | 25 | 42 | 23 | **0.045** | 7 | 7 | 21 | 26 | 39 | 5 | 9 | 33 | 28 | 25 | **0.015** |
|  | Knowledge | 10 | 13 | 16 | 21 | 39 | 9 | 12 | 36 | 26 | 17 | **≤0.001** | 11 | 14 | 16 | 19 | 40 | 2 | 15 | 29 | 40 | 15 | 0.180 | 10 | 13 | 18 | 22 | 37 | 10 | 12 | 37 | 23 | 18 | **0.001** |
| ID | Importance | 0 | 1 | 5 | 21 | 72 | 0 | 1 | 7 | 31 | 60 | **0.003** | 0 | 0 | 4 | 19 | 77 | 0 | 2 | 17 | 25 | 56 | **0.003** | 1 | 2 | 6 | 23 | 69 | 0 | 1 | 5 | 33 | 61 | 0.134 |
|  | Influence | 16 | 18 | 19 | 21 | 25 | 16 | 19 | 31 | 25 | 9 | **0.015** | 2 | 1 | 9 | 33 | 56 | 4 | 6 | 6 | 44 | 40 | 0.053 | 27 | 31 | 27 | 14 | 2 | 18 | 22 | 36 | 21 | 3 | **0.001** |
|  | Resources | 4 | 4 | 18 | 29 | 45 | 5 | 12 | 27 | 38 | 18 | **≤0.001** | 1 | 1 | 7 | 36 | 56 | 0 | 4 | 2 | 54 | 40 | 0.107 | 6 | 7 | 26 | 25 | 36 | 6 | 13 | 32 | 35 | 14 | **≤0.001** |
|  | Knowledge | 5 | 6 | 28 | 40 | 21 | 5 | 14 | 29 | 37 | 14 | **0.002** | 0 | 0 | 19 | 52 | 29 | 0 | 8 | 6 | 52 | 33 | 0.533 | 8 | 11 | 34 | 32 | 15 | 6 | 15 | 35 | 34 | 10 | 0.272 |
| T | Importance | 0 | 0 | 1 | 16 | 84 | 0 | 1 | 6 | 38 | 54 | **≤0.001** | 0 | 0 | 2 | 11 | 88 | 0 | 0 | 0 | 48 | 52 | **≤0.001** | 0 | 0 | 0 | 19 | 81 | 0 | 1 | 7 | 36 | 55 | **≤0.001** |
|  | Influence | 41 | 12 | 11 | 15 | 20 | 48 | 20 | 14 | 8 | 10 | **0.001** | 2 | 2 | 14 | 35 | 47 | 0 | 4 | 10 | 31 | 54 | 0.419 | 69 | 20 | 9 | 1 | 1 | 59 | 24 | 15 | 3 | 0 | **0.018** |
|  | Resources | 7 | 6 | 20 | 24 | 44 | 23 | 13 | 25 | 21 | 17 | **≤0.001** | 1 | 1 | 11 | 29 | 59 | 0 | 2 | 6 | 42 | 50 | 0.471 | 12 | 9 | 27 | 20 | 32 | 29 | 16 | 29 | 16 | 10 | **≤0.001** |
|  | Knowledge | 15 | 17 | 35 | 22 | 10 | 27 | 21 | 26 | 18 | 8 | **≤0.001** | 1 | 11 | 36 | 35 | 17 | 0 | 2 | 21 | 54 | 23 | **0.006** | 26 | 22 | 34 | 12 | 6 | 33 | 25 | 27 | 10 | 5 | 0.051 |
|  | Support Colleagues* | 9 | 8 | 30 | 28 | 25 | 16 | 11 | 34 | 20 | 10 | **0.011** | 1 | 6 | 17 | 39 | 37 | 0 | 2 | 21 | 40 | 33 | 0.659 | 16 | 10 | 40 | 19 | 15 | 19 | 13 | 37 | 15 | 5 | 0.611 |
|  | Support Supervisor* | 16 | 12 | 28 | 22 | 23 | 26 | 13 | 34 | 8 | 7 | **0.001** | 6 | 10 | 21 | 28 | 34 | 10 | 2 | 38 | 17 | 23 | 0.812 | 23 | 13 | 33 | 18 | 13 | 30 | 15 | 34 | 6 | 4 | 0.144 |
| IC | Importance | 0 | 0 | 4 | 12 | 84 | 0 | 1 | 5 | 20 | 74 | **≤0.001** | 0 | 0 | 7 | 15 | 78 | 0 | 0 | 15 | 35 | 50 | **≤0.001** | 0 | 0 | 1 | 11 | 88 | 0 | 2 | 6 | 27 | 65 | **≤0.001** |
|  | Influence | 2 | 5 | 20 | 32 | 41 | 6 | 10 | 19 | 37 | 28 | **≤0.001** | 0 | 9 | 28 | 33 | 29 | 0 | 17 | 25 | 33 | 25 | 0.378 | 3 | 2 | 14 | 31 | 50 | 7 | 9 | 18 | 37 | 29 | **≤0.001** |
|  | Resources | 2 | 4 | 11 | 30 | 54 | 2 | 5 | 14 | 45 | 33 | **≤0.001** | 1 | 3 | 14 | 30 | 52 | 0 | 2 | 23 | 50 | 25 | **0.006** | 2 | 4 | 9 | 29 | 55 | 3 | 6 | 13 | 44 | 35 | **≤0.001** |
|  | Knowledge | 0 | 3 | 15 | 40 | 41 | 2 | 4 | 19 | 44 | 30 | **0.002** | 0 | 4 | 22 | 40 | 33 | 0 | 2 | 23 | 50 | 25 | 0.600 | 1 | 3 | 10 | 41 | 46 | 3 | 5 | 19 | 43 | 31 | **≤0.001** |
|  | Support Colleagues* | 2 | 9 | 23 | 36 | 31 | 1 | 6 | 16 | 46 | 29 | 0.130 | 1 | 10 | 22 | 38 | 30 | 0 | 8 | 23 | 46 | 19 | 0.875 | 2 | 8 | 23 | 35 | 31 | 2 | 6 | 14 | 46 | 31 | 0.116 |
|  | Support Supervisor* | 7 | 14 | 22 | 32 | 25 | 4 | 9 | 24 | 30 | 30 | **0.015** | 7 | 16 | 23 | 30 | 24 | 4 | 8 | 38 | 23 | 23 | 0.611 | 7 | 13 | 20 | 34 | 26 | 4 | 9 | 21 | 32 | 32 | **0.039** |
| Note. All numbers shown are percentages, except from p-values; these are p-values of the Mann-Whitney U-test: Asymptotic Sig. (2-sided test).  SD=Screening Diagnostics, ID=Infection diagnosis, T=Treatment, IC=Infection control, DE=Germany, NL=The Netherlands  *49 Dutch respondents in hospital #3 could choose Not Applicable for questions about support by colleagues/supervisor. If NA was chosen, responses were handled as missing value (excluded from Mann-Whitney U tests) for these questions | | | | | | | | | | | | | | | | | | | | | | | | | | | | | | | | | | |
