# Supplementary material for: Cross-border comparison of antimicrobial resistance (AMR) and AMR prevention measures: the healthcare workers’ perspective
Source: Antimicrob Resist Infect Control. 2019 Jul 22;8:123. doi: 10.1186/s13756-019-0577-4 (PMC6647090; doi:10.1186/s13756-019-0577-4)
Supplement: Supplementary file 3 — Table S2b. Ranked ANCOVA (corrected for age, gender and years of hospital experience) results of (i) all respondents, (ii) German/Dutch physicians, and (iii) German/Dutch nurses, including p-values of differences between nationalities. Table S3b. Ranked ANCOVA (corrected for age, gender and years of hospital experience) results of (i) all respondents, (ii) German/Dutch physicians, and (iii) German/Dutch nurses, including p-values of differences between nationalities. Comparison of unadjusted (Mann-Whitney U tests) adjusted (ranked ANCOVA (corrected for age, gender and years of hospital experience)) results. (DOCX 32 kb) [file 13756_2019_577_MOESM3_ESM.docx]

## Additional file 3

**Title:**

Table S2b. Statement ranked ANCOVA (corrected for age, sex and years of hospital experience) results of (i) all respondents, (ii) German/Dutch physicians, and (iii) German/Dutch nurses, including p-values of differences between nationalities.

Table 3b. AMSS ranked ANCOVA (corrected for age, sex and years of hospital experience) results of (i) all respondents, (ii) German/Dutch physicians, and (iii) German/Dutch nurses, including p-values of differences between nationalities.

**Description:**

Comparison of unadjusted (Mann-Whitney U tests) and adjusted (ranked ANCOVA (corrected for age, sex and years of hospital experience)) results.

## Table S2b. Statement ranked ANCOVA (corrected for age, sex and years of hospital experience) results of (i) all respondents, (ii) German/Dutch physicians, and (iii) German/Dutch nurses, including p-values of differences between nationalities.

| ***Statements*** | | **All respondents (n=574)** | | | | | | **Physicians (n=177)** | | | | | | **Nurses (n=397)** | | | | | |
| --- | --- | --- | --- | --- | --- | --- | --- | --- | --- | --- | --- | --- | --- | --- | --- | --- | --- | --- | --- |
|  |  | Mann-Whitney | | | ANCOVA* | | | Mann-Whitney | | | ANCOVA* | | | Mann-Whitney | | | ANCOVA* | | |
|  |  | **DE** (n=305) | **NL** (n=269) | P-value | **DE** (n=305) | **NL** (n=269) | P-value | **DE** (n=305) | **NL** (n=269) | P-value | **DE** (n=305) | **NL** (n=269) | P-value | **DE** (n=305) | **NL** (n=269) | P-value | **DE** (n=305) | **NL** (n=269) | P-value |
|  |  | Mean rank | Mean rank |  | Mean rank | Mean rank |  | Mean rank | Mean rank |  | Mean rank | Mean rank |  | Mean rank | Mean rank |  | Mean rank | Mean rank |  |
| AMR is a problem for … | the general population. | 256.04 | 323.17 | ≤0.001 | 257.52 | 321.49 | ≤0.001 | 84.28 | 101.33 | 0.026 | 85.38 | 98.45 | ***0.109*** | 170.42 | 221.99 | ≤0.001 | 170.77 | 221.72 | ≤0.001 |
|  | nursing homes. | 287.73 | 287.24 | 0.968 | 293.97 | 280.17 | 0.286 | 88.61 | 90.03 | 0.851 | 89.46 | 87.79 | 0.207 | 200.00 | 198.20 | 0.860 | 204.73 | 194.39 | 0.331 |
|  | our hospital. | 276.31 | 300.19 | 0.043 | 279.34 | 296.76 | ***0.166*** | 86.19 | 96.34 | 0.180 | 86.41 | 95.76 | 0.784 | 192.99 | 203.84 | 0.262 | 193.84 | 203.16 | 0.36 |
|  | my patients. | 269.51 | 307.89 | 0.002 | 270.74 | 306.50 | 0.006 | 86.66 | 95.10 | 0.281 | 86.48 | 95.59 | 0.282 | 185.90 | 209.54 | 0.017 | 185.66 | 209.73 | 0.021 |
| One of the leading causes of AMR is… | the use of antibiotics in farming animals. | 359.76 | 205.57 | ≤0.001 | 363.50 | 201.33 | ≤0.001 | 95.18 | 72.87 | 0.004 | 96.95 | 68.24 | ≤0.001 | 266.27 | 144.88 | ≤0.001 | 266.72 | 144.52 | ≤0.001 |
|  | the use of antibiotics by patients. | 271.68 | 305.43 | 0.011 | 273.27 | 303.63 | 0.033 | 91.55 | 82.34 | 0.254 | 92.09 | 80.93 | 0.196 | 212.52 | 188.12 | 0.027 | 208.80 | 191.11 | ***0.126*** |
|  | the admission of nursing home patients. | 304.33 | 268.42 | 0.006 | 300.93 | 272.27 | 0.041 | 83.69 | 102.88 | 0.021 | 83.63 | 103.02 | 0.03 | 193.60 | 203.34 | 0.380 | 194.20 | 202.86 | 0.458 |
| I believe that… | antibiotics are prescribed at the request of patients. | 317.27 | 253.75 | ≤0.001 | 315.35 | 255.93 | ≤0.001 | 94.76 | 73.95 | 0.013 | 95.99 | 70.74 | 0.005 | 220.26 | 181.90 | 0.001 | 217.85 | 183.83 | 0.004 |
|  | antibiotic prescriptions should be based on lab results. | 329.38 | 240.02 | ≤0.001 | 326.81 | 242.93 | ≤0.001 | 96.87 | 68.45 | ≤0.001 | 97.03 | 68.03 | ≤0.001 | 233.78 | 171.02 | ≤0.001 | 230.34 | 173.79 | ≤0.001 |
|  | I am sufficiently informed about the diagnostic policy. | 306.63 | 265.81 | 0.002 | 314.04 | 257.41 | ≤0.001 | 89.61 | 87.41 | 0.791 | 91.33 | 82.91 | 0.304 | 217.39 | 184.21 | 0.003 | 222.68 | 179.95 | ≤0.001 |
|  | broad spectrum antibiotics should be provided when there is doubt of an infection. | 250.72 | 329.21 | ≤0.001 | 252.35 | 327.36 | ≤0.001 | 81.82 | 107.77 | 0.001 | 97.22 | 67.54 | ≤0.001 | 175.51 | 217.90 | ≤0.001 | 176.05 | 217.46 | ≤0.001 |
|  | I can contribute sufficiently to limit AMR. | 337.63 | 230.66 | ≤0.001 | 331.28 | 237.86 | ≤0.001 | 100.09 | 60.03 | ≤0.001 | 101.30 | 56.88 | ≤0.001 | 224.66 | 178.36 | ≤0.001 | 225.79 | 177.44 | ≤0.001 |
| *Ranked ANOVA corrected for age, sex and years of hospital experience  Results that are not significantly different in the adjusted analyses are marked italic/bold and red. | | | | | | | | | | | | | | | | | | | |

## Table S3b. AMSS ranked ANCOVA (corrected for age, sex and years of hospital experience) results of (i) all respondents, (ii) German/Dutch physicians, and (iii) German/Dutch nurses, including p-values of differences between nationalities.

| ***APM*** | | All respondents (n=574) | | | | | | Physicians (n=177) | | | | | | Nurses (n=397) | | | | | | |
| --- | --- | --- | --- | --- | --- | --- | --- | --- | --- | --- | --- | --- | --- | --- | --- | --- | --- | --- | --- | --- |
|  |  | Mann-Whitney | | | ANCOVA* | | | Mann-Whitney | | | ANCOVA* | | | Mann-Whitney | | | ANCOVA* | | | |
|  |  | DE (n=305) | NL (n=269) | P-value | DE (n=305) | NL (n=269) | P-value | DE (n=305) | NL (n=269) | P-value | DE (n=305) | NL (n=269) | P-value | DE (n=305) | NL (n=269) | P-value | | DE (n=305) | NL (n=269) | P-value |
|  |  | Mean rank | Mean rank |  | Mean rank | Mean rank |  | Mean rank | Mean rank |  | Mean rank | Mean rank |  | Mean rank | Mean rank |  |  | Mean rank | Mean rank |  |
| Screening diagnostics | Importance | 309.77 | 261.26 | ≤0.001 | 316.78 | 253.35 | ≤0.001 | 95.03 | 73.24 | 0.002 | 95.77 | 71.31 | 0.001 | 218.18 | 182.76 | ≤0.001 | | 221.52 | 180.09 | ≤0.001 |
|  | Feeling sufficiently equipped | 308.15 | 264.08 | 0.001 | 312.13 | 259.58 | ≤0.001 | 92.09 | 80.93 | 0.192 | 92.88 | 78.86 | 0.104 | 212.24 | 188.35 | 0.038 | | 216.48 | 184.94 | 0.009 |
| Infection diagnosis | Importance | 303.73 | 269.10 | 0.003 | 310.73 | 261.16 | ≤0.001 | 94.42 | 74.84 | 0.004 | 95.39 | 72.32 | 0.001 | 207.09 | 192.49 | 0.134 | | 212.27 | 188.33 | 0.018 |
|  | Feeling sufficiently equipped | 316.19 | 254.97 | ≤0.001 | 305.84 | 266.71 | 0.005 | 91.91 | 81.41 | 0.214 | 91.56 | 82.32 | 0.304 | 202.26 | 196.38 | 0.609 | | 204.17 | 194.84 | 0.438 |
| Treatment | Importance | 328.20 | 241.35 | ≤0.001 | 333.19 | 235.69 | ≤0.001 | 97.26 | 67.43 | ≤0.001 | 98.69 | 63.70 | ≤0.001 | 229.79 | 174.23 | ≤0.001 | | 233.82 | 170.99 | ≤0.001 |
|  | Feeling sufficiently equipped | 320.02 | 250.63 | ≤0.001 | 306.19 | 266.31 | 0.003 | 86.97 | 94.31 | 0.392 | 87.20 | 93.70 | 0.471 | 213.17 | 187.60 | 0.027 | | 211.03 | 189.33 | 0.073 |
| Infection control | Importance | 317.55 | 253.43 | ≤0.001 | 323.95 | 246.17 | ≤0.001 | 96.19 | 70.21 | ≤0.001 | 97.52 | 66.74 | ≤0.001 | 225.11 | 177.99 | ≤0.001 | | 228.10 | 175.59 | ≤0.001 |
|  | Feeling sufficiently equipped | 295.76 | 278.14 | 0.202 | 305.87 | 266.67 | 0.007 | 90.92 | 83.99 | 0.419 | 91.01 | 83.75 | 0.428 | 209.70 | 190.39 | 0.094 | | 215.38 | 185.82 | 0.014 |
| *Ranked ANOVA corrected for age, sex and years of hospital experience | | | | | | | | | | | | | | | | | | | | |
